# Complication and Sequelae of COVID-19: What Should We Pay Attention to in the Post-Epidemic Era

**DOI:** 10.3389/fimmu.2021.711741

**Published:** 2021-09-03

**Authors:** Keda Yang, Guangfu Wen, Jinpeng Wang, Siming Zhou, Wacili Da, Yan Meng, Yuchuan Xue, Lin Tao

**Affiliations:** ^1^Department of Orthopedics, First Hospital of China Medical University, Shenyang, China; ^2^Department of Pediatrics, Shengjing Hospital of China Medical University, Shenyang, China; ^3^The First Department of Clinical Medicine, China Medical University, Shenyang, China

**Keywords:** COVID-19, complication, sequelae, eight systems, bibliometric analysis, Web of Science

## Abstract

COVID-19 is widespread worldwide and seriously affects the daily life and health of humans. Countries around the world are taking necessary measures to curb the spread. However, COVID-19 patients often have at least one organ complication and sequelae in addition to respiratory symptoms. Controlling the epidemic is only a phased victory, and the complication and sequelae of COVID-19 will need more attention in the post-epidemic era. We collected general information from over 1000 articles published in 2020 after the COVID-19 outbreak and systematically analyzed the complication and sequelae associated with eight major systems in COVID-19 patients caused by ACE2 intervention in the RAS regulatory axis. The autoimmune response induced by 2019-nCoV attacks and damages the normal tissues and organs of the body. Our research will help medical workers worldwide address COVID-19 complication and sequelae.

## Introduction

COVID-19 was one of the most concerning issues in 2020. Coronavirus threatens the life and health of humans (1). In addition, no country around the world has been completely spared. At present, humans have not truly controlled the spread of 2019-nCoV, and due to the alteration in climate, a second epidemic has appeared. According to the data of the World Health Organization (WHO), the global number of COVID-19 cases has reached to over 130 million, with no sign of weakening. Our previous studies indicated that 2019-nCoV and SARS share a very similar viral gene sequence, origin, and intracellular receptor, ACE2 (2). People infected with 2019-nCoV will have symptoms of varying degrees ranging from a fever or a slight cough to pneumonia and even death (3). Most patients are asymptomatic or have mild cases, and even patients with more severe symptoms can recover with the help of medical treatment.

A prominent problem in the post-epidemic era is the complication and sequelae of COVID-19. All systems of the human body are at risk of being attacked by COVID-19 (4). A number of studies have demonstrated that patients with 2019-nCoV have at least one organ complication and sequelae in addition to respiratory symptoms even if the symptoms are mild (5, 6). More than three-quarters of COVID-19 patients reported having at least one sequelae six months after the onset of the disease (7). Although COVID-19 is an infectious disease characterized by pneumonia, complication affecting the cardiovascular system are the most serious and fatal. In clinical testing of COVID-19 patients, it was found that the level of angiotensin converting enzyme (ACE) was extremely low (8). A decline in ACE increases the risk of hypertension, myocarditis, and even heart failure (9–11). Meanwhile, the impact of 2019-nCoV on the nervous system is also very serious. A study indicated that nearly 40% of COVID-19 patients showed neurological symptoms (12). Severe encephalitis causes cerebral parenchymal disease and induces brain death (13). In addition, there is a certain impact of 2019-nCoV on the digestive, urinary and endocrine systems (14–16). Comparative genomics of different strains of 2019-nCoV around the world have been fully analyzed (17–19). As more and more people in various countries are vaccinated, it is believed that the epidemic will be brought under control soon. The prevention of and interventions for patients’ complication and sequelae will be the focus in the post-epidemic era.

Bibliometric analysis is an interdisciplinary science which could obtain the objective and quantifiable data of certain field (20). It could provide research hotspots and directions in a specific field by analyzing various parameters of published works and foster collaborative transdisciplinary research (21, 22). Therefore, this study aimed to explore the research status and to predict future trends of COVID-19 complication and sequelae by using original research bibliometric analysis. We believe our study will attract the attention of medical workers and researchers worldwide and enrich the process of COVID-19 prevention and management.

## Materials and Methods

### Search Strategy and Inclusion Criteria

The relevant articles were retrieved from the Web of Science core collection database according to the indexes COVID-19 and complication or sequelae on December 30, 2020. The time range of retrieval covered all years, that is, from the year that the database was created to the search date. There were a total of 1169 publications, and their types included reviews, articles, early access, editorial materials, and letters. Then, we established the inclusion criteria and screened the articles against the inclusion criteria: (a) published in English, (b)involved COVID-19 research, and (c) focused on a discussion of complication and sequelae (**Supplementary Figure 1**).

### Data Extraction

The above operations were performed by two reviewers independently, and a third reviewer helped them build a consensus when disagreements occurred. The general information of these articles was listed, including the title, publication date, name of the first author and corresponding author, geographic origin, publication journal, publication institution, research theme, and journal impact factor.

### Bibliometric Analysis

The extracted information of these articles was imported into the Online Analysis Platform of Literature Metrology (http://bibliometric.com/) and integrated according to their characteristics. CiteSpace V5.5.R1 SE, 64 bit (Drexel University, Philadelphia, PA, USA) and VOSviewer (Leiden University, Leiden, the Netherlands) were used to visualize the network of this information, such as authors and counties, institutions, and journals (23, 24). We also identified research trends and predicted evolutionary directions in terms of high-frequency keywords on CiteSpace. The approach is described in detail in our previous study on COVID-19 and the SARS and MERS coronaviruses (2).

## Results

### Most Active Countries and Institutions

**Figure 1:**
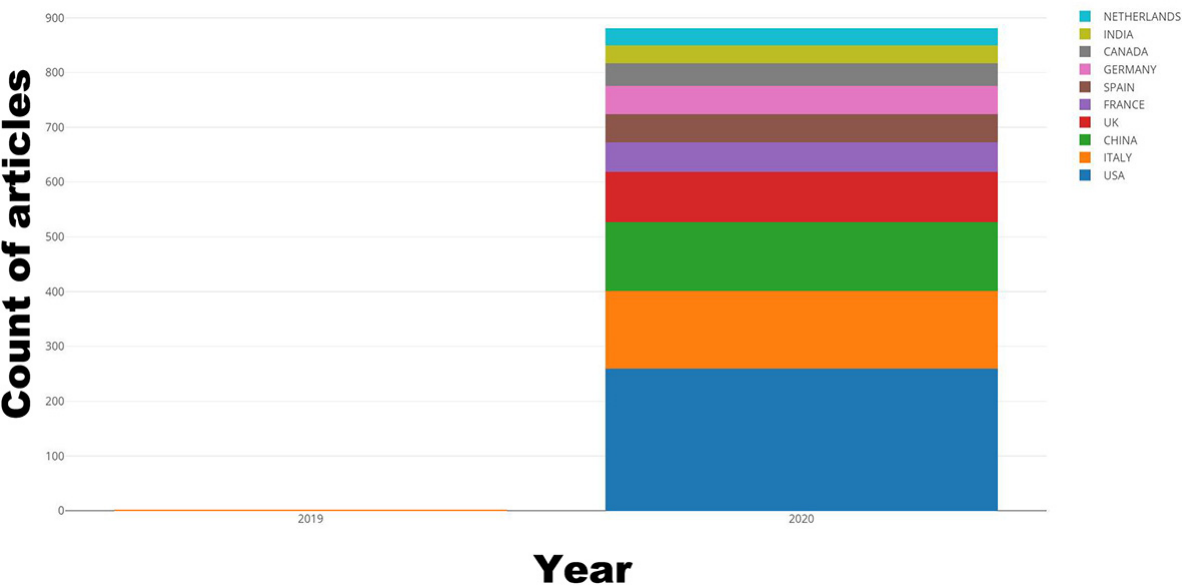
The distribution of countries/regions. The development of the top 10 countries/regions.

**Figure 2:**
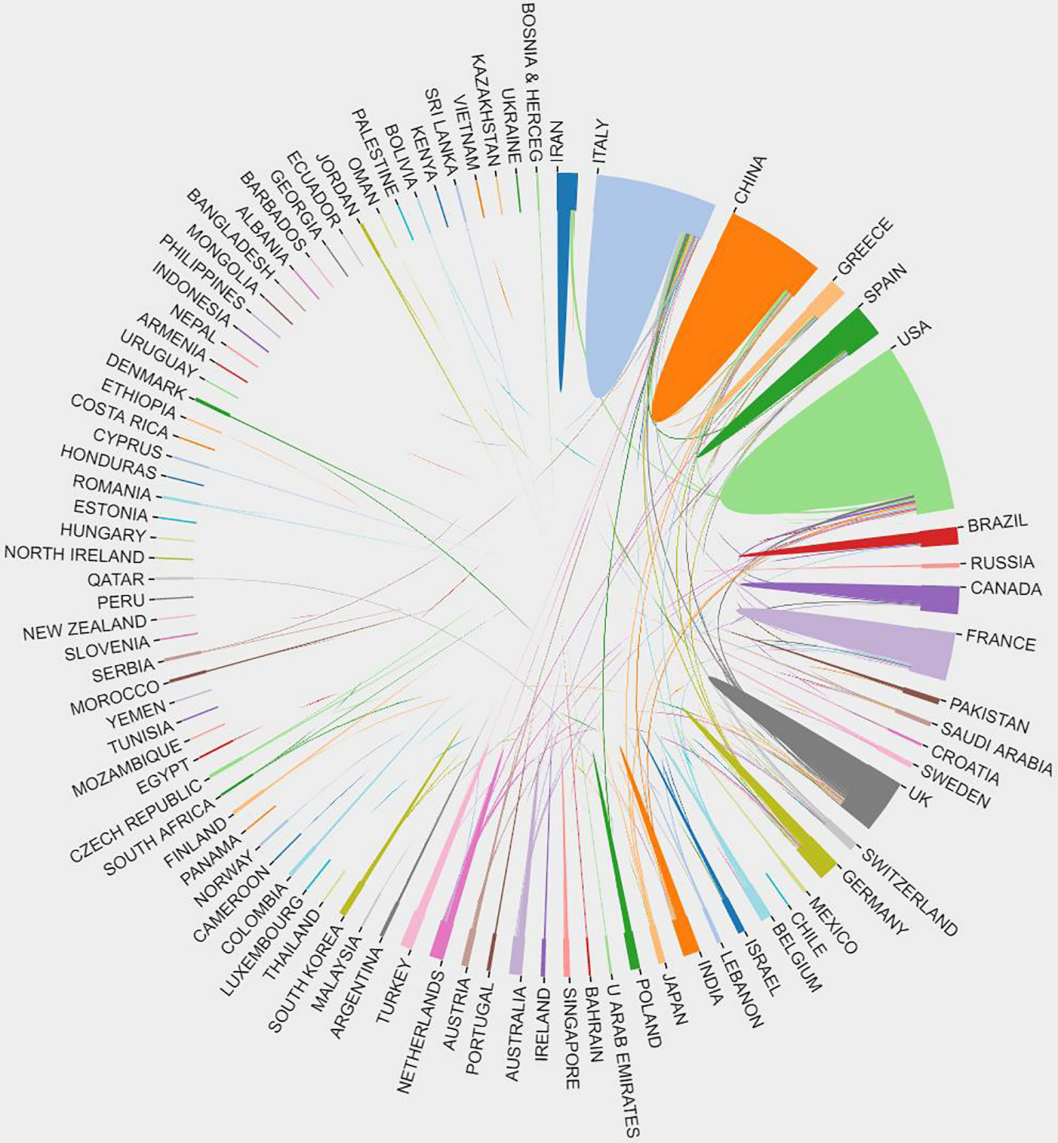
The distribution of countries/regions. The cooperation of countries/regions.

**Figure 3:**
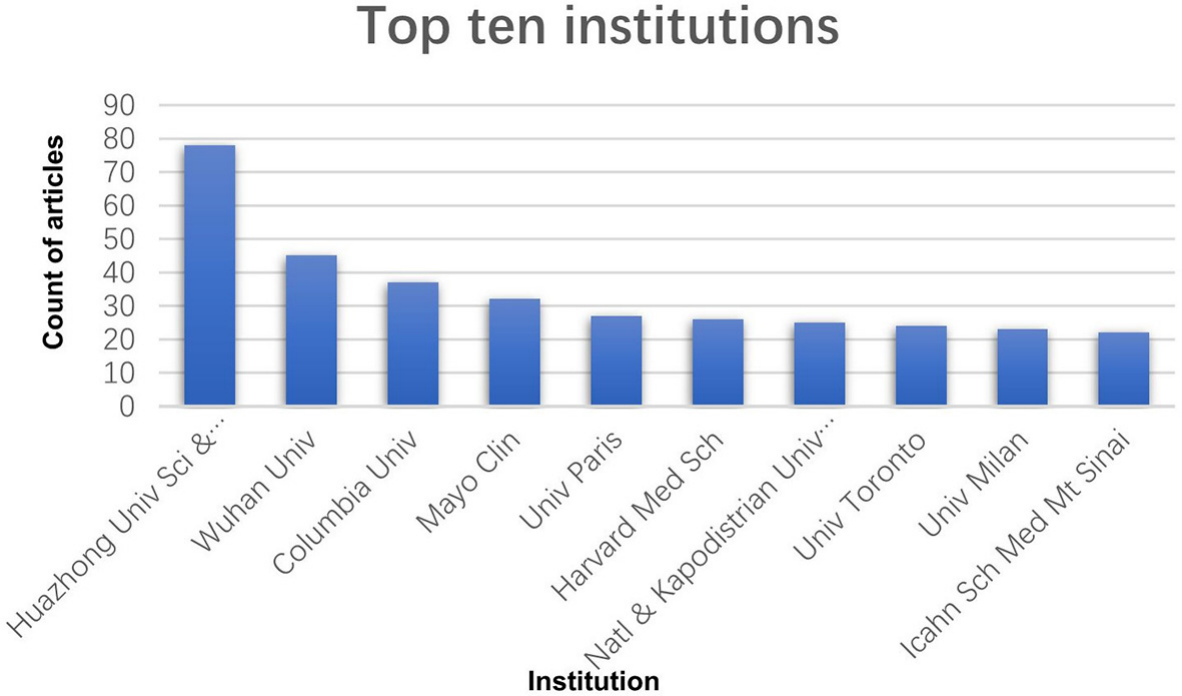
The top ten institutions ranked in terms of the article counts.

The top ten most active countries according to the number of publications each year are shown in **Figure 1**. These ten countries all have highly developed economies and dense population flows, resulting in wide and rapid spread of the epidemic. Unsurprisingly, America and European countries (especially the UK) were the predominant countries. China also contributed a large number of articles due to the early detection of the coronavirus. International cooperation was dominated by the United States, Italy, and China, which was consistent with the ranking by the number of publishing articles (**Figure 2**). Based on the number of articles, we listed the top 10 institutions (**Figure 3**). Two universities from Wuhan, China, ranked in the top two. Huazhong University of Science and Technology ranked first with 78 articles, and Wuhan University ranked second with 45 articles. Columbia University, the Mayo Clinic and the University of Paris ranked third, fourth and fifth, with 37, 32 and 27 articles, respectively. Besides, Harvard Medical School, the National and Kapodistrian University of Athens, the University of Toronto, the University of Milan, and the Icahn School of Medicine at Mount Sinai each published over 20 articles.

### Most Active Authors and Journals

**Figure 4:**
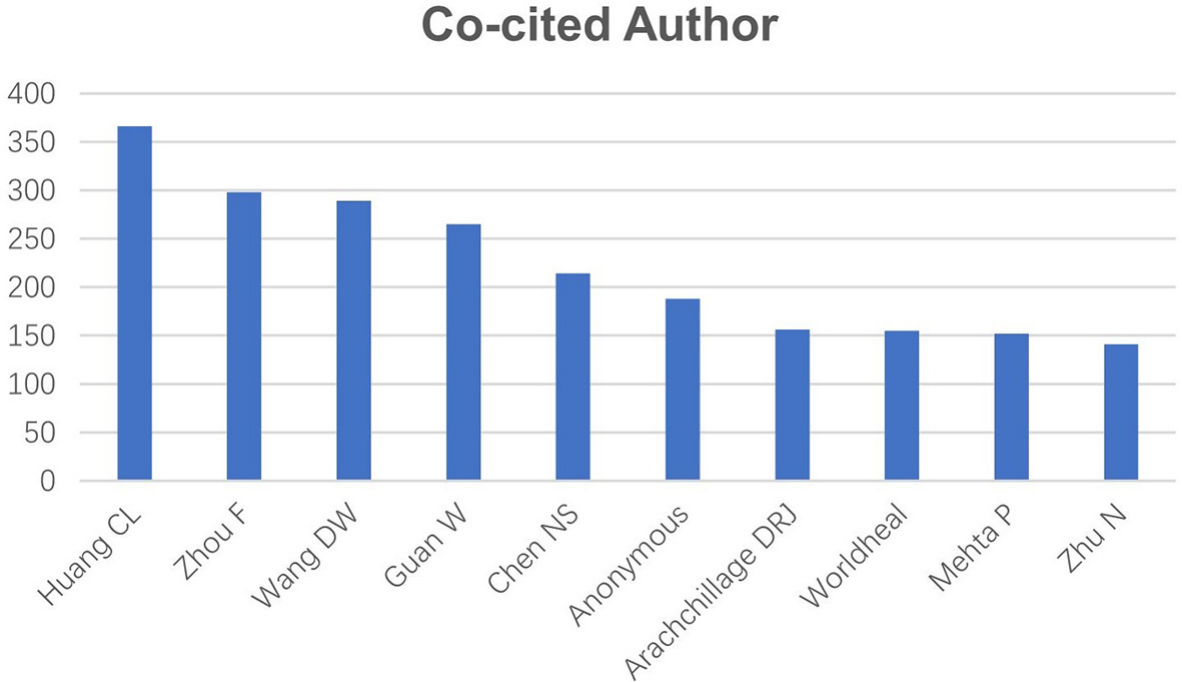
The distribution of authors involved in COVID-19 complication and sequelae research. The article counts of the top ten co-cited authors.

**Figure 5:**
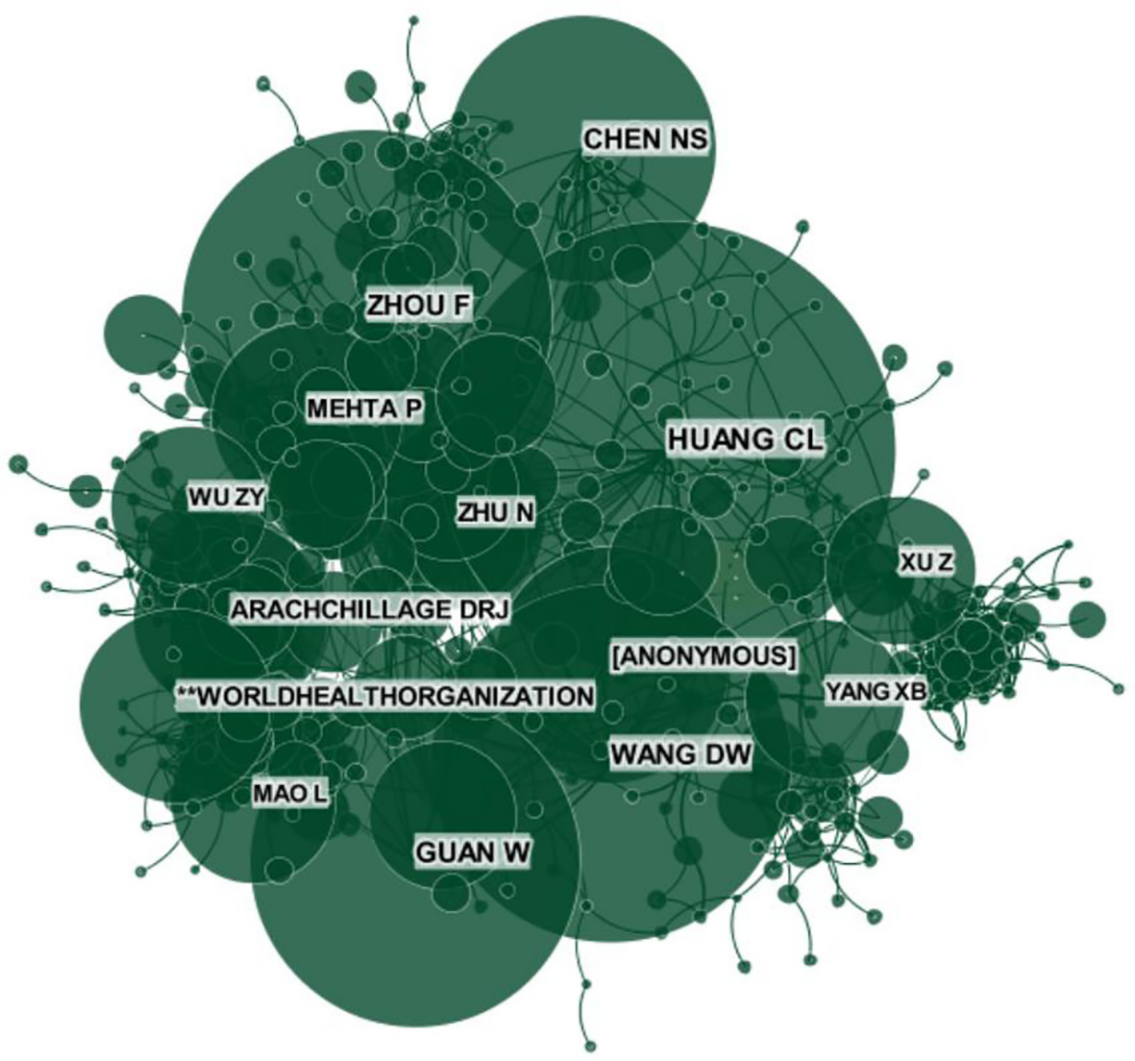
The distribution of authors involved in COVID-19 complication and sequelae research. The network map of co-cited authors.

We counted the authors who were the first or corresponding authors of these articles and ranked them in descending order by the published article count for the top ten authors (**Table 1**). Seven of these authors were from China. Zhang, Y published the most articles (n=8), one as the first author. Liu, H ranked second with five articles. Each of the remaining eight authors published 4 articles each: Wang, L, Connors, JM, Thachil, J, Khan, S, Liu, W, Li, XY, Yang, F and Chen, T. The top ten co-cited authors are listed in **Figure 4** in descending order of article count. CiteSpace was used to reveal the network of these co-cited authors (**Figure 5**). All of these authors participated in over 100 articles and in over 200 articles of the top five authors. They were Huang CL (n=366), Zhou F (n=298), Wang DW (n=289), Guan W (n=265) and Chen NS (n=214).

**Table 1:** The top ten authors contributed to the research about COVID-19 complication and sequelae sorted by total number of articles.

Rank	Author	Article counts	First author counts	Corresponding author counts
1	Zhang, Y	8	1	0
2	Liu, H	5	1	0
3	Wang, L	4	1	1
4	Connors, JM	4	1	1
5	Thachill, J	4	2	1
6	Khan, S	4	1	0
7	Liu, W	4	1	1
8	Li, XY	4	2	0
9	Yang, F	4	2	0
10	Chen, T	4	1	0

The top ten journals are listed in descending order by the number of published articles (**Table 2**). The *Journal of Medical Virology* (IF: 2.327) published 14 articles, which was the highest number and accounted for 14.4% of the total 97 articles published by the ten journals. The second ranked journal was *Journal of Clinical Medicine* (IF: 4.241), which published 12 articles. Ten articles were published in each of the next four journals. They were *Journal of Thrombosis and Haemostasis* (IF: 5.824), *Journal of Stroke and Cerebrovascular Disease* (IF: 2.136), *Frontiers in Neurology* (IF: 4.003) and *Diabetes Research and Clinical Care* (IF: 5.602). The total citation count of *Journal of Thrombosis and Haemostasis* (IF: 5.824) was 85, belonging to Q1 according to the quartile in category which represents the credibility and recognition of the journal. In addition, of all these journals, *Critical Care* has the highest impact factors (IF: 9.097), while *Frontiers in Immunology* ranks the second (IF: 7.561).

**Table 2:** The top 10 journals that published articles in COVID-19 complication and sequelae research sorted by article counts.

Rank	Journal	Article counts	Total counts of citation	Impact factor	Quartile in Category
1	Journal of Medical Virology	14	32	2.327	Q3
2	Journal of Clinical Medicine	12	1	4.241	Q1
3	Journal of Thrombosis and Haemostasis	10	85	5.824	Q1
4	Journal of Stroke and Cerebrovascular Diseases	10	5	2.136	Q2
5	Frontiers in Neurology	10	1	4.003	Q3
6	Diabetes Research and Clinical Practice	10	1	5.602	Q1
7	Critical Care	9	5	9.097	Q1
8	Frontiers in Immunology	8	1	7.561	Q2
9	Clinical Medicine	7	4	2.659	Q1
10	European Review for Medical and Pharmacological Sciences	7	0	3.507	Q2

### Research Hotspots

**Figure 6:**
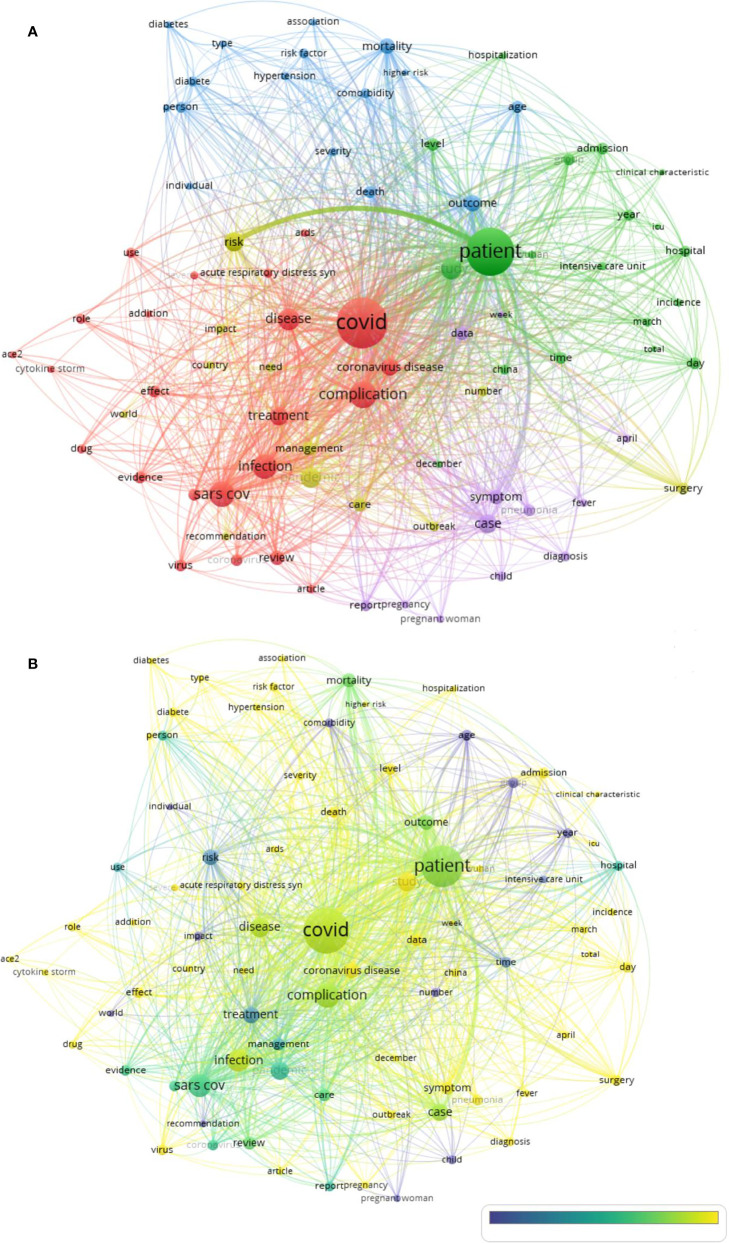
Keywords in publications about COVID-19 complication and sequelae research. **(A)** Mapping of the keywords into seven clusters with different colors. **(B)** Distribution of keywords was presented according to the appearance for the average time.

A total of 82 keywords were extracted and are listed in **Table 3**. The number of occurrences of keywords ranged from 69 to 3972 times. Through VOSviewer analysis, these items were classified into 5 clusters, which are shown in **Figures 6A, B**. Each cluster had a common theme: cluster 1 (23 items, in red, treatment), cluster 2 (19 items, in green, clinical manifestation), cluster 3 (16 items, in blue, risk factors), cluster 4 (12 items, in yellow, morbidity), and cluster 5 (12 items, in purple, diagnosis). The top five keywords of each cluster are listed as follows, and the number of occurrences is indicated:

Cluster 1: COVID (3972), Complication (1196), SARS cov (1028), Infection (970), Disease (761)
Cluster 2: Patient (3249), Study (685), Level (280), Time (231), Admission (227).
Cluster 3: Outcome (417), Mortality (363), Death (237), Age (226), Person (190).
Cluster 4: Pandemic (619), Risk (490), Management (314), Care (281), Surgery (234).
Cluster 5: Case (555), Symptom (352), Data (294), Pneumonia (216), Report (193).

**Table 3:** The analytic consequence of 82 keywords with from 69 to 3972 occurrence times.

Keywords	cluster	Links	Total link strength	Occurrences	Average publishing years	Average citations
Ace2	1	77	2950	82	20.3049	2.1854
Acute Respiratory Distress Syndrome	1	81	5067	127	26.063	2.8052
Addition	1	81	3290	89	3.7528	0.4039
Admission	2	81	11509	227	10.6123	1.1422
Age	3	81	11724	226	17.2522	1.8612
April	5	81	6073	121	6.3471	0.6831
Ards	1	77	4060	102	12.5294	1.3486
Article	1	81	4303	122	4.4016	0.4738
Association	3	81	5055	93	30.5484	3.288
Care	4	81	9407	281	5.0641	0.5468
Case	5	81	21451	555	12.6649	1.364
Child	5	81	4932	151	4.0464	0.4355
China	2	81	7650	172	25.407	2.7346
Clinical Characteristic	2	81	4076	77	32.6104	3.5099
Comorbidity	3	81	8449	178	19.7584	2.1349
Complication	1	81	43447	1196	9.8712	1.0629
Coronavirus	1	81	7972	232	15.3578	1.6551
Coronavirus Disease	1	81	19632	498	18.998	2.0448
Country	4	81	4056	108	10	1.0763
Covid	1	81	136572	3972	10.1803	1.0958
Cytokine Storm	1	78	2548	81	5.9012	0.6352
Data	5	81	14373	294	15.0272	1.6174
Day	2	81	10904	215	17.8326	1.9193
Death	3	81	11838	237	16.8608	1.8148
December	2	81	3254	81	21.679	2.3333
Diabete	3	81	7891	173	6.948	0.7478
Diabetes	3	79	5083	89	6.1461	0.6615
Diagnosis	5	81	6494	157	8.9873	0.9673
Disease	1	81	29238	761	11.2943	1.2156
Drug	1	81	5427	144	8.0764	0.8693
Effect	1	81	8792	222	9.4324	1.0152
Evidence	1	81	8986	241	11.1452	1.1996
Fever	5	81	4561	108	18.3796	1.9782
Group	2	81	11325	222	11.8288	1.2732
Higher Risk	3	81	3236	73	17.8356	1.9197
Hospital	2	80	9604	180	16.0056	1.7254
Hospitalization	2	81	4762	95	24.3789	2.6239
Hypertension	3	81	5960	111	21.8739	2.3543
Icu	2	79	3409	69	11.2174	1.2073
Impact	4	81	6252	164	10.7988	1.1683
Incidence	2	81	5196	102	10.3922	1.1185
Individual	3	81	3734	97	6.5773	0.713
Infection	1	81	36677	970	10.5299	1.1339
Intensive Care Unit	2	81	5042	109	10.7339	1.1598
Level	2	81	11449	280	28.3036	3.0464
Management	4	81	11873	314	6.9841	0.7548
March	2	80	7026	142	8.0845	0.8701
Mortality	3	81	18373	363	18.1488	1.9547
Need	4	81	4283	126	7.5635	0.8141
Number	4	81	7299	170	8.6294	0.9317
Obesity	3	80	4120	89	4.382	0.4716
Outbreak	4	81	7508	162	20.1605	2.1699
Outcome	3	81	20998	417	16.8585	1.8157
Pandemic	4	81	21700	619	5.2763	0.5687
Patient	2	81	125336	3249	16.9471	1.8251
Person	3	81	7936	190	5.5632	0.5988
Pneumonia	5	81	8778	216	13.3704	1.4391
Pregnancy	5	80	5430	114	19.5088	2.0998
Pregnant Woman	5	79	4581	102	13.4314	1.4456
Recommendation	4	80	4012	116	7.9224	0.8527
Report	5	81	7448	193	14.399	1.5523
Review	1	81	12982	327	7.2263	0.7778
Risk	4	81	18852	490	9.4224	1.0182
Risk Factor	3	81	7551	152	9.0724	0.9765
Role	1	81	5383	171	3.6433	0.3921
Sars Cov	1	81	36063	1028	9.2091	0.9926
Severe Acute Respiratory Syndrome Coronavirus	1	81	8304	232	13.431	1.4477
Severe Covid	1	81	4092	103	6.0485	0.651
Severity	3	81	6534	139	11.0504	1.1894
Study	2	81	32033	685	12.8307	1.381
Surgery	4	78	10541	234	7.6325	0.8215
Symptom	5	81	13853	352	12.054	1.2974
Time	2	81	9549	231	13.8701	1.4971
Total	2	80	3852	77	16.6883	1.7962
Treatment	1	81	19442	521	8.3129	0.8985
Type	3	81	5512	96	10.0417	1.0808
Use	1	81	5959	154	9.3247	1.0068
Virus	1	81	8190	234	9.3761	1.0092
Week	5	79	3737	74	26.8649	2.8915
World	4	81	2941	84	6.6071	0.717
Wuhan	2	81	4830	105	34.2762	3.6892
Year	2	81	10295	200	24.045	2.588
Ace2	1	77	2950	82	20.3049	2.1854
Acute Respiratory Distress Syndrome	1	81	5067	127	26.063	2.8052
Addition	1	81	3290	89	3.7528	0.4039
Admission	2	81	11509	227	10.6123	1.1422
Age	3	81	11724	226	17.2522	1.8612
April	5	81	6073	121	6.3471	0.6831
Ards	1	77	4060	102	12.5294	1.3486
Article	1	81	4303	122	4.4016	0.4738
Association	3	81	5055	93	30.5484	3.288
Care	4	81	9407	281	5.0641	0.5468
Case	5	81	21451	555	12.6649	1.364
Child	5	81	4932	151	4.0464	0.4355
China	2	81	7650	172	25.407	2.7346
Clinical Characteristic	2	81	4076	77	32.6104	3.5099
Comorbidity	3	81	8449	178	19.7584	2.1349
Complication	1	81	43447	1196	9.8712	1.0629
Coronavirus	1	81	7972	232	15.3578	1.6551
Coronavirus Disease	1	81	19632	498	18.998	2.0448
Country	4	81	4056	108	10	1.0763
Covid	1	81	136572	3972	10.1803	1.0958
Cytokine Storm	1	78	2548	81	5.9012	0.6352
Data	5	81	14373	294	15.0272	1.6174
Day	2	81	10904	215	17.8326	1.9193
Death	3	81	11838	237	16.8608	1.8148
December	2	81	3254	81	21.679	2.3333
Diabete	3	81	7891	173	6.948	0.7478
Diabetes	3	79	5083	89	6.1461	0.6615
Diagnosis	5	81	6494	157	8.9873	0.9673
Disease	1	81	29238	761	11.2943	1.2156
Drug	1	81	5427	144	8.0764	0.8693
Effect	1	81	8792	222	9.4324	1.0152
Evidence	1	81	8986	241	11.1452	1.1996
Fever	5	81	4561	108	18.3796	1.9782
Group	2	81	11325	222	11.8288	1.2732
Higher Risk	3	81	3236	73	17.8356	1.9197
Hospital	2	80	9604	180	16.0056	1.7254
Hospitalization	2	81	4762	95	24.3789	2.6239
Hypertension	3	81	5960	111	21.8739	2.3543
Icu	2	79	3409	69	11.2174	1.2073
Impact	4	81	6252	164	10.7988	1.1683
Incidence	2	81	5196	102	10.3922	1.1185
Individual	3	81	3734	97	6.5773	0.713
Infection	1	81	36677	970	10.5299	1.1339
Intensive Care Unit	2	81	5042	109	10.7339	1.1598
Level	2	81	11449	280	28.3036	3.0464
Management	4	81	11873	314	6.9841	0.7548
March	2	80	7026	142	8.0845	0.8701
Mortality	3	81	18373	363	18.1488	1.9547
Need	4	81	4283	126	7.5635	0.8141
Number	4	81	7299	170	8.6294	0.9317
Obesity	3	80	4120	89	4.382	0.4716
Outbreak	4	81	7508	162	20.1605	2.1699
Outcome	3	81	20998	417	16.8585	1.8157
Pandemic	4	81	21700	619	5.2763	0.5687
Patient	2	81	125336	3249	16.9471	1.8251
Person	3	81	7936	190	5.5632	0.5988
Pneumonia	5	81	8778	216	13.3704	1.4391
Pregnancy	5	80	5430	114	19.5088	2.0998
Pregnant Woman	5	79	4581	102	13.4314	1.4456
Recommendation	4	80	4012	116	7.9224	0.8527
Report	5	81	7448	193	14.399	1.5523
Review	1	81	12982	327	7.2263	0.7778
Risk	4	81	18852	490	9.4224	1.0182
Risk Factor	3	81	7551	152	9.0724	0.9765
Role	1	81	5383	171	3.6433	0.3921
Sars Cov	1	81	36063	1028	9.2091	0.9926
Severe Acute Respiratory Syndrome Coronavirus	1	81	8304	232	13.431	1.4477
Severe Covid	1	81	4092	103	6.0485	0.651
Severity	3	81	6534	139	11.0504	1.1894
Study	2	81	32033	685	12.8307	1.381
Surgery	4	78	10541	234	7.6325	0.8215
Symptom	5	81	13853	352	12.054	1.2974
Time	2	81	9549	231	13.8701	1.4971
Total	2	80	3852	77	16.6883	1.7962
Treatment	1	81	19442	521	8.3129	0.8985
Type	3	81	5512	96	10.0417	1.0808
Use	1	81	5959	154	9.3247	1.0068
Virus	1	81	8190	234	9.3761	1.0092
Week	5	79	3737	74	26.8649	2.8915
World	4	81	2941	84	6.6071	0.717
Wuhan	2	81	4830	105	34.2762	3.6892
Year	2	81	10295	200	24.045	2.588
Ace2	1	77	2950	82	20.3049	2.1854
Acute Respiratory Distress Syndrome	1	81	5067	127	26.063	2.8052
Addition	1	81	3290	89	3.7528	0.4039
Admission	2	81	11509	227	10.6123	1.1422
Age	3	81	11724	226	17.2522	1.8612
April	5	81	6073	121	6.3471	0.6831
Ards	1	77	4060	102	12.5294	1.3486
Article	1	81	4303	122	4.4016	0.4738
Association	3	81	5055	93	30.5484	3.288
Care	4	81	9407	281	5.0641	0.5468
Case	5	81	21451	555	12.6649	1.364
Child	5	81	4932	151	4.0464	0.4355
China	2	81	7650	172	25.407	2.7346
Clinical Characteristic	2	81	4076	77	32.6104	3.5099
Comorbidity	3	81	8449	178	19.7584	2.1349
Complication	1	81	43447	1196	9.8712	1.0629
Coronavirus	1	81	7972	232	15.3578	1.6551
Coronavirus Disease	1	81	19632	498	18.998	2.0448
Country	4	81	4056	108	10	1.0763
Covid	1	81	136572	3972	10.1803	1.0958
Cytokine Storm	1	78	2548	81	5.9012	0.6352
Data	5	81	14373	294	15.0272	1.6174
Day	2	81	10904	215	17.8326	1.9193
Death	3	81	11838	237	16.8608	1.8148
December	2	81	3254	81	21.679	2.3333
Diabete	3	81	7891	173	6.948	0.7478
Diabetes	3	79	5083	89	6.1461	0.6615
Diagnosis	5	81	6494	157	8.9873	0.9673
Disease	1	81	29238	761	11.2943	1.2156
Drug	1	81	5427	144	8.0764	0.8693
Effect	1	81	8792	222	9.4324	1.0152
Evidence	1	81	8986	241	11.1452	1.1996
Fever	5	81	4561	108	18.3796	1.9782
Group	2	81	11325	222	11.8288	1.2732
Higher Risk	3	81	3236	73	17.8356	1.9197
Hospital	2	80	9604	180	16.0056	1.7254
Hospitalization	2	81	4762	95	24.3789	2.6239
Hypertension	3	81	5960	111	21.8739	2.3543
Icu	2	79	3409	69	11.2174	1.2073
Impact	4	81	6252	164	10.7988	1.1683
Incidence	2	81	5196	102	10.3922	1.1185
Individual	3	81	3734	97	6.5773	0.713
Infection	1	81	36677	970	10.5299	1.1339
Intensive Care Unit	2	81	5042	109	10.7339	1.1598
Level	2	81	11449	280	28.3036	3.0464
Management	4	81	11873	314	6.9841	0.7548
March	2	80	7026	142	8.0845	0.8701
Mortality	3	81	18373	363	18.1488	1.9547
Need	4	81	4283	126	7.5635	0.8141
Number	4	81	7299	170	8.6294	0.9317
Obesity	3	80	4120	89	4.382	0.4716

## Discussion

So far, 2019-nCoV has infected one hundred and thirty million people and affected the lives of billions of people. As the understanding of COVID-19 patients continues to develop, clinicians have gradually realized the dangers of the sequelae and complication that COVID-19 patients have. Early detection of possible complication and sequelae of each system will improve clinical outcomes and reduce their incidence. Laboratory and clinical data will be crucial to clarify the pathophysiology and potential damage of 2019-nCoV to various systems. Finally, the various system evaluations of patients after recovery are critical to understanding the natural history of 2019-nCoV in various systems and monitoring potential system sequelae. Therefore, we took the eight major systems as the entry point and analyzed in detail the morbidity and pathogenesis of the possible complication and sequelae of each system of COVID-19 patients (**Supplementary Figure 2**).

### Respiratory Sequelae

The respiratory system is the primary target for attack by 2019-nCoV. Pathological changes occurred in the pulmonary parenchyma, pulmonary vascular endothelium, and alveoli of patients infected with COVID-19 (25). There are case reports that the alternations in pulmonary parenchyma caused by 2019-nCoV could induce the occurrence of pneumothorax, leading to severe vasculitis and endotheliitis (26). In the tissue section observations of the lung tissue of patients who died from COVID-19, it was found that the alveoli were destroyed and infiltrated by a large number of inflammatory cells, and the pulmonary microvascular was accompanied by a large number of thromboses (27, 28). Additionally, many patients recovering from COVID-19 still have a cough and shortness of breath after being cured, and severe cases can have extensive fibrosis of the lungs, resulting in severe dyspnea (29). The above alternations would cause an imbalance of the ventilatory flow ratio, and there is no evidence that these pathological alterations are reversible. Therefore, patients infected with COVID-19 are impacted with severe pulmonary fibrosis and are still at risk after their nucleic acid test turns negative.

### Cardiovascular Complication

Similar to other respiratory infections, pre-existing cardiovascular disease or risk factors increase susceptibility to COVID-19 (30). Meanwhile, the mortality of patients with cardiovascular disease is relatively higher (31). The cardiovascular diseases of patients infected with 2019-nCoV worsen significantly, and it can even cause new cardiovascular complication. A study from Wuhan, China showed that nearly 20% of patients infected with 2019-nCoV suffered cardiovascular damage and the patients’ conditions would worsen if their IL-6 was elevated (32, 33). The most serious cardiovascular complication of COVID-19 patients is myocarditis, which is most commonly defined as an elevation of hypersensitivity cardiac troponin I (cTnI) above the 99^th^ percentile of the upper reference limit (34). The onset of acute myocarditis results in ischemia and hypoxia and increases the risk of death if it exceeds the bearing capacity of the body (35, 36). In addition, both tachy- and brady-arrhythmias are known to occur in COVID-19 patients (37). However, no study has described the incidence of ST-segment elevation myocardial infarction in COVID-19, but it appears to be low. Angiotensin converting enzyme 2 (ACE2) plays an essential role in the development of cardiovascular diseases (38). High expression of ACE2 in COVID-19 patients lead to an imbalance of renin-angiotensin-aldosterone system (RAAS), which participates in the regulation of electrolytes and blood flow (38, 39). Excessive contraction of blood vessels and acceleration of blood flow increased the risk of hypertension and thrombosis (40). In addition, elevated pressure in the peripheral blood increases the afterload on the heart and consequently causes organic pathological alterations of cardiac dilatational hypertrophy (41). A study by Frankfurt University Hospital showed that 78 out of 100 survivors of COVID-19 had cardiovascular abnormalities, and 60 of them still had signs of persistent myocardial inflammation more than two months after the diagnosis (42).

### Neurological Complication

Neurological symptoms are also common in patients infected with 2019-nCoV (43, 44). Nearly forty percent of patients have neurological manifestations (12). Most of these effects are seizures, delirium, and unconsciousness (45–47). A British study analyzed 153 patients with clinical syndromes related to COVID-19 pneumonia. Many of the patients suffered from new-onset psychosis, neurocognitive syndromes or affective disorders, and changes in mental status, which usually occurred in relatively young patients. Some showed symptoms associated with Guillain-Barre syndrome (48–50). When this virus infects the brain, viral encephalitis and purulent lesions increase the difficulty of treatment and caused sequelae after treatment (51). In addition, the most dangerous condition was that the virus would induce paralysis of the respiratory center, causing apnea and threatening the patient’s life if it invaded the medulla oblongata and pons (44). Recent studies indicated that 2019-nCoV can break through the blood-brain barrier and invade the brain, mainly through the nasal cavity and intestines and the virus has been detected in cerebrospinal fluid (52–54). In addition, it was found in the clinical examination of patients with neurological symptoms that almost all patients on imaging had brain bleeding to different degrees (55). Cranial nerve injury leads to a loss of smell, temporary loss of consciousness, and even stroke (56–58). Local hypoxemia induced by cerebral circulation disorders can lead to permanent alterations of the brain (12).

### Digestive Complication

The clinical findings indicated that gastrointestinal reactions and liver dysfunction appeared in patients with COVID-19 (59, 60). Inappetence, vomiting, diarrhea and indigestion occurred in the patients. A meta-analysis of patient data from multiple countries revealed that more than half of patients had gastrointestinal reactions (14). In addition, in a retrospective analysis of clinical cases, it was reported that 27.3% of patients showed liver biochemistry abnormalities and 3.9% had liver injury (61). The disturbance of the digestive system is attributed to the expression of ACE2 and the ACE2 receptor in digestive ducts (62, 63). The pathogenesis is similar to the complication experienced in the cardiovascular systems. However, most of the gastrointestinal complication caused by COVID-19 disappeared after effective treatment of the pneumonia, with few obvious sequelae. In addition, more noteworthy was the role of intestinal flora in the development of COVID-19. On the one hand, clinical data indicated the intestinal flora was disordered in patients with COVID-19, which lead to the adverse reactions in the gastrointestinal tract (64, 65). On the other hand, the intestinal flora had the potential to promote the activity of anti-inflammation cells in the alveolar and inhibit the progression of pneumonia, which is defined as the “gut-lung axis” (66).

### Urinary Complication

The kidney is one of the important target organs causing the high mortality rate of COVID-19 (67). A clinical study entitled “Caution on Kidney Dysfunctions of 2019-nCoV Patients” published on the preprint platform medRxiv stated that 63% of patients showed symptoms of renal insufficiency, and CT scans showed 100% of patients with renal imaging abnormalities. It has been noted that kidney involvement in COVID-19 is frequent and it ranges from mild proteinuria to acute kidney injury (AKI) (68, 69). Numerous studies have indicated a high incidence of AKI in patients with COVID-19, especially in severe cases (70–72). According to clinical statistics, the mortality rate of COVID-19 patients with AKI was three times higher than in patients without kidney injury (73). Microscopic observation of the kidneys of patients who died of COVID-19 found that most patients had obvious renal tubular damage (74). Acute kidney injury significantly contributes to long-term effects on the kidney, such as microalbuminuria and chronic kidney diseases or renal failure, which needs routine dialysis (75). However, no studies have been reported on the long-term renal outcomes among patients recovered from COVID-19 (76). Therefore, it is essential to monitor the renal function of patients who are discharged from the hospital, especially with kidney damage caused by coronaviruses infection in order to improve the prognosis of COVID-19 and to prevent further renal injuries.

The kidney injury was more due to the secondary effect of immune reactions induced by 2019-nCoV rather than any direct effect of the virus (77). In other words, although COVID-19 could invade the kidney cells through the ACE2-dependent pathway, the virus-associated immune response and cytokine secretion were more likely to attack normal cells and cause kidney damage (78). However, this is based on only a consensus of the majority, and the pathogenesis of COVID-19 in kidney damage is still inconclusive. Identifying the reason for the kidney alterations will contribute to increased efficiency of treatment and an improved prognosis.

### Endocrine Complication

In terms of the clinical manifestations of patients with COVID-19, most endocrine glands might be affected by the virus, such as the pancreas, adrenal glands, thyroid, etc. (79). Recent studies have indicated that patients with a history of diabetes were more susceptible to 2019-nCoV (80, 81). In addition, the presence of diabetes mellitus increased the risk of requiring critical care (82, 83). It was found in a microscope observation of the adrenal glands of patients with COVID-19 that 46% showed alterations in adrenal gland morphology such as ischemia, hemorrhage, degeneration, and local inflammation (84). Adrenal vascular alterations are likely to be caused by the immune disorder induced by 2019-nCoV (85). In addition, 15% of patients with COVID-19 had thyroid dysfunction (86). This was also attributed to the immunity response and autoimmunity induced by 2019-nCoV (87, 88). Meanwhile, the outbreak of the COVID-19 epidemic might worsen some glandular tumors and delay the timing of treatment (89). The above evidence showed that 2019-nCoV could attack the glands *via* the immune system, which might be the entry point for the treatment of endocrine complication. If there is no effective treatment, it will further aggravate the condition of diabetes and increase the risk of cancer and other chronic endocrine diseases.

### Reproductive Complication

There are obvious sex differences in reproductive complication and sequelae. Recent studies had confirmed ACE2 is abundant in gametocytes, Sertoli cells and spermatogonia stem cells, suggesting the testis is a high-risk organ vulnerable to 2019-nCoV infection (90, 91). Indeed, coronavirus could damage the structure of the testicles and affect male fertility, supported by the detection of coronavirus in the semen of patients, some of whom had complication of orchitis (92). An observational study indicated that 39.1% of male patients with COVID-19 have oligospermia and 60.9% of them have a high inflammatory state in their semen (93). Around 19% of patients at the time of their COVID-19 confirmation were experiencing scrotal discomfort (94). In addition, interstitial oedema and congestion in the testes and epididymis are observed in all deceased COVID-19 patients, which also indicate potential testis injury (93). Compared with male patients, there are relatively few reports on the impact of the coronavirus on the female reproductive system. However, ACE2 has been demonstrated to be widely expressed in the female genital tract and ovary (95, 96), and thereby is likely to affect ovulation and embryonic development in a similar way (97, 98). Due to a lack of attention and limited duration of hospitalization, other reproductive dysfunctions such as infertility and menstrual disorders are difficult to identify. Therefore, follow-up studies and evaluation of reproductive function, especially fertility, is essential among patients recovered from COVID-19.

### Skeletal Complication

During the epidemic of SARS, myalgia and muscle weakness were common complication (99). COVID-19 also has a direct impact on the skeletal system together with an indirect action through the regulation of nerves and the blood supply. 2019-nCoV combines with ACE2 and destroys the balance of RAS, which causes degradation of muscle protein and leads to physical dysfunction (100). In addition, infection-mediated deleterious immune responses lead to neurological abnormalities, and a significant increase in serum pro-inflammatory cytokines may lead to skeletal muscle damage (101). In addition, patients with myalgia tend to have higher levels of creatine kinase and lactate dehydrogenase (12). Elevated levels of the pro-inflammatory factor interleukin-6 may be responsible for the myalgia and joint pain (102). Osteoimmunology reveals the potential effect of viral infection on the RANK-RANKL system, which increases bone resorption and induces osteoporosis. It is worth noting that after being discharged from the hospital, severe patients may have sequelae of muscle wasting due to the prolonged period spent in bed, and they require progressive rehabilitation training to gradually return to their pre-onset mobility (103). Furthermore, severe patients treated with glucocorticoids may also experience hip joint injuries and femoral head necrosis (104). At present, it seems that the patient’s condition is not effectively controlled, and an overdose of glucocorticoids is given, which may cause sequelae such as osteoporosis and osteonecrosis.

### Other Involvement: Psychological Changes

The influence of COVID-19 on mental health due to restricted activity in public should not be ignored. The mental health status reported by patients with COVID-19 appears to be more serious. They fear death and are very prone to negative emotions (105). Unlike SARS survivors, the incidence of depression, anxiety, insomnia, and acute stress in patients with COVID-19 is 75%, 71%, 68%, and 71%, respectively, while the incidence of mental and psychological problems in COVID-19 survivors, 3 months after discharge and 30 months after discharge, is also much higher.

There are two aspects to the psychological complication or sequelae caused by COVID-19. One is that severely ill patients have experienced the fear of death. Even if their bodies recover, some people still have psychological fears. The other is that patients with mild illnesses cause people to fear rejection by others after their recovery. Many people who have recovered from mild illness have nervousness, anxiety, and fear. Regardless of whether they had mild or severe cases, nearly half of survivors have post-traumatic stress disorder, depression, or insomnia and anxiety. Some people cannot return to a normal state.

The mental and psychological problems of medical staff are even more prominent, and the incidence is significantly higher than that of non-medical staff. Among them, nurses have the highest incidence of psychological problems. It is indicated that anxiety among medical staff is 46%, while the incidence among non-medical staff is 8.5%. Other people, including front-line workers and their families, close contacts, and people in isolation, as well as low-income people in the community, are also susceptible to psychological problems, indicating that ordinary citizens also experience poor psychological health in the epidemic. It is shown that in the first five months of the pandemic, Japan’s monthly suicide rate dropped by 14%, but it rose by 16% in the second wave 90, while the suicide rate of women, children, and adolescents has increased even more.

Therefore, in the post-epidemic era, the construction of a mental health system is particularly important. A grid-based community psychological service system should be established, especially in the prevention stage. For example, a three-person psychological service team should be formed in schools, communities and some medical units to screen and serve for the mental health of the population. In addition, mental and psychological education courses should also be introduced in primary and secondary schools to avoid impairments of the mental development of the students.

As is mentioned above, clinical complications and sequelae of COVID-19 were common among survivors during early convalescence. Apart from the clinical characteristics during hospitalization, the outcomes of COVID-19 also vary across different ages and ethnic backgrounds. It was observed in a large cohort of survivors of COVID-19 in Wuhan, China that survivors with old age are especially susceptible to complications or sequelae of COVID-19 compared with younger individuals (106). Indeed, ageing plays an important role in the development of COVID-19 and its complications or sequelae. With age, mitochondrial quality, and function declines, leading to a variety of physiology changes, such as chronic inflammation and defective immune responses to viral infection, which eventually increases the susceptibility to COVID-19 complications and sequelae (107, 108). Likewise, the outcomes of COVID-19 also present certain ethnic and racial differences. It was observed that black patients with COVID-19 usually had higher mortality rates that white patients, which was also noted among pediatric patients. However, the racial disparities are more like to be multifactorial while black race alone was not associated with the high mortality rates (109–111). Black patients had higher prevalence of chronic diseases, such as diabetes, hypertension, chronic renal diseases and so forth. As is mentioned above, patients with pre-existing diseases usually had bad poor clinical outcomes and were with higher risks of various complications and sequelae. Additionally, work environment, living conditions, public insurance and economic conditions were also responsible for the prevalence and mortality rate of COVID-19 (110).

Currently, no specific treatment targeting major complications and sequelae of COVID-19, which is only limited to symptomatic treatment. Additionally, there is even no effective therapy for complications in certain systems, such as the reproductive system. Therefore, current treatment options should be extended in patients with COVID-19 and personalized therapies should be implemented based on the clinical characteristics during hospitalization and also the individual circumstances, such as age and pre-existing diseases. Consequently, it is crucial to development reasonable and effective treatment strategies to restore function of organs which were damaged during the course of COVID. In addition, daily symptom monitoring is necessary for patients discharged from hospital. Routine physical examination is strongly recommended for severe or elderly patients and patients with pre-existing diseases until 6 months after discharge.

There are several limitations that are specific to the use of bibliometric analysis in this study which may be inevitable. It has been only less than two years since the outbreak of COVID-19. Although significant breakthroughs have been made in the understanding COVID-19, there are relatively few literatures focused on the complications and sequelae of COVID-19 that have been published. As a result, only 82 keywords were extracted from the published literatures, which may lead to the inaccuracies in the classification of keywords. In addition, with accumulating evidence indicating that many survivors of COVID-19 are suffered from various complications and sequelae, it is believed that growing numbers of studies will focus on the complications and sequelae of COVID-19. Therefore, there may be discrepancies between our bibliometric analysis and the actual publication situation.

## Conclusions

In the context of vaccination and effective medications, there are no obstacles to the elimination of 2019-nCoV. However, the damage of 2019-nCoV to the body has been ignored. We systematically analyzed the complication and sequelae of the eight systems of COVID-19 patients. Most of the complication or sequelae of patients are related to the expression of ACE2 on tissues and organs, which is the target recognized and bound by 2019-nCoV. The RAS axis participates in the regulatory process and leads to systemic disorders when the axis feedback is imbalanced. At the same time, we analyzed various psychological barriers of people in the post-epidemic era. In our study, we pay more attention to the adverse variations to the body caused by 2019-nCoV rather than just the virus itself. In conclusion, the prevention and treatment of complications and sequelae in COVID-19 patients in the post-epidemic era are the top priority, and they should and must attract the close attention of experts and scholars around the world.

## Author Contributions

Conceptualization: LT, KY, JW, and GW. Methodology: KY, GW, and SZ. Investigation: LT, KY, GW, JW, SZ, WD, YM, and YX. Writing – original draft: KY, GW, JW, SZ, WD, YM, and YX. Writing – review and editing: LT, KY, JW, and GW. Supervision: LT, KY, JW, and GW. All authors contributed to the article and approved the submitted version.

## Funding

This work was supported by the fellowship of China Postdoctoral Science Foundation [2020M670100ZX].

## Conflict of Interest

The authors declare that the research was conducted in the absence of any commercial or financial relationships that could be construed as a potential conflict of interest.

## Publisher’s Note

All claims expressed in this article are solely those of the authors and do not necessarily represent those of their affiliated organizations, or those of the publisher, the editors and the reviewers. Any product that may be evaluated in this article, or claim that may be made by its manufacturer, is not guaranteed or endorsed by the publisher.

## Supplementary Material

Supplementary Figure 1 Flow chart of literature filtering involved in this study.

Supplementary Figure 2 Complication and sequelae of COVID-19 patients involved in eight major systems.

## References

[1] Moore B Lee P Hewish M Dixon B Mukherjee T. Coronaviruses in Training Centre for Intellectually Retarded. Lancet (London England) (1977) 1:261. 10.1016/S0140-6736(77)91064-9 PMC7134688 64794

[2] Tao Z Zhou S Yao R Wen K Da W Meng Y. COVID-19 Will Stimulate a New Coronavirus Research Breakthrough: A 20-Year Bibliometric Analysis. Ann Trans Med (2020) 8:528. 10.21037/atm.2020.04.26 PMC7214912 32411751

[3] Wu F Zhao S Yu B Chen YM Wang W Song ZG. A New Coronavirus Associated With Human Respiratory Disease in China. Nature (2020) 579:265–9. 10.1038/s41586-020-2008-3 PMC7094943 32015508

[4] Jamwal S Gautam A Elsworth J Kumar M Chawla R Kumar P. An Updated Insight Into the Molecular Pathogenesis, Secondary Complications and Potential Therapeutics of COVID-19 Pandemic. Life Sci (2020) 257:118105. 10.1016/j.lfs.2020.118105 32687917 PMC7366108

[5] Beghi E Helbok R. The EAN COVID-19 Registry (ENERGY): An International Instrument for Surveillance of Neurological Complications in Patients With COVID-19. Euro J Neurol (2020). 10.1111/ene.14652 PMC7753513 33220127

[6] Zahra W Dixon JW Mirtorabi N Rolton DJ Tayton ER Hale PC. Safety Evaluation of a Strategy to Restart Elective Orthopaedic Surgery During the De-Escalation Phase of the COVID-19 Pandemic. Bone Joint Open (2020) 1:450–6. 10.1302/2633-1462.18.BJO-2020-0105.R1 PMC7667222 33215138

[7] Huang C Huang L Wang Y Li X Ren L Gu X. 6-Month Consequences of COVID-19 in Patients Discharged From Hospital: A Cohort Study. Lancet (London England) (2021) 397:220–32. 10.1016/S0140-6736(20)32656-8 PMC7833295 33428867

[8] South AM Tomlinson L Edmonston D. Controversies of Renin-Angiotensin System Inhibition During the COVID-19 Pandemic. Nat Rev Nephrol (2020) 16:305–7. 10.1038/s41581-020-0279-4 PMC7118703 32246101

[9] Campbell WG Jr. Donohue JA Duket LH. Serum Angiotensin Converting Enzyme Activity and the Capacity to Develop Hypertention-Associated Arterial Disease. Studies During the Induction Phase of One-Kidney Perinephritis Hypertension in Rabbits. Am J Pathol (1978) 93:383–404. 213977 PMC2018369

[10] Souza-Silva TG Vilas-Boas DF Gonçalves-Santos E Mazzeti AL Caldas IS Gonçalves RV. Impact of Diminazene Aceturate on Renin-Angiotensin System, Infectious Myocarditis and Skeletal Myositis in Mice: An *In Vitro* and *In Vivo* Study. Life Sci (2020) 257:118067. 10.1016/j.lfs.2020.118067 32652140

[11] Santema BT Ouwerkerk W Tromp J Sama IE Ravera A Regitz-Zagrosek V. Identifying Optimal Doses of Heart Failure Medications in Men Compared With Women: A Prospective, Observational, Cohort Study. Lancet (London England) (2019) 394:1254–63. 10.1016/S0140-6736(19)31792-1 31447116

[12] Mao L Jin H Wang M Hu Y Chen S He Q. Neurologic Manifestations of Hospitalized Patients With Coronavirus Disease 2019 in Wuhan, China. JAMA Neurol (2020) 77:683–90. 10.1001/jamaneurol.2020.1127 PMC7149362 32275288

[13] Cao A Rohaut B Guennec LL Saheb S Marois C Altmayer V. Severe COVID-19-Related Encephalitis Can Respond to Immunotherapy. Brain: J Neurol (2020). 143(12):e102. 10.1093/brain/awaa337 PMC7665364 33064794

[14] Cheung KS Hung IFN Chan PPY Lung KC Tso E Liu R. Gastrointestinal Manifestations of SARS-CoV-2 Infection and Virus Load in Fecal Samples From a Hong Kong Cohort: Systematic Review and Meta-Analysis. Gastroenterol (2020) 159:81–95. 10.1053/j.gastro.2020.03.065 PMC7194936 32251668

[15] Rabaan AA Al-Ahmed SH Sah R Tiwari R Yatoo MI Patel SK. SARS-CoV-2/COVID-19 and Advances in Developing Potential Therapeutics and Vaccines to Counter This Emerging Pandemic. Ann Clin Microbiol Antimicrob (2020) 19:40. 10.1186/s12941-020-00384-w 32878641 PMC7464065

[16] Alshareef R Al Zahrani A Alzahrani A Ghandoura L. Impact of the COVID-19 Lockdown on Diabetes Patients in Jeddah, Saudi Arabia. Diabetes Metab Syndrome (2020) 14:1583–7. 10.1016/j.dsx.2020.07.051 PMC7422800 32947759

[17] Zhang YZ Holmes EC. A Genomic Perspective on the Origin and Emergence of SARS-CoV-2. Cell (2020) 181:223–7. 10.1016/j.cell.2020.03.035 PMC7194821 32220310

[18] Gudbjartsson DF Helgason A Jonsson H Magnusson OT. Spread of SARS-CoV-2 in the Icelandic Population. New Engl J Med (2020) 382:2302–15. 10.1101/2020.03.26.20044446 PMC7175425 32289214

[19] Oude Munnink BB Nieuwenhuijse DF. Rapid SARS-CoV-2 Whole-Genome Sequencing and Analysis for Informed Public Health Decision-Making in the Netherlands. Nature Med (2020) 26:1405–10. 10.1038/s41591-020-0997-y 32678356

[20] Guo Y Hao Z Zhao S Gong J Yang F. Artificial Intelligence in Health Care: Bibliometric Analysis. J Med Internet Res (2020) 22:e18228. 10.2196/preprints.18228 32723713 PMC7424481

[21] Noyons ECM Moed HF Luwel M. Combining Mapping and Citation Analysis for Evaluative Bibliometric Purposes: A Bibliometric Study. J Assoc Inf Ence Technol (2010) 50:14652.

[22] Niu B Hong S Yuan J Peng S Wang Z Zhang X. Global Trends in Sediment-Related Research in Earth Science During 1992–2011: A Bibliometric Analysis. Scientometrics (2014) 98:511–29. 10.1007/s11192-013-1065-x

[23] Zhang T Yin X Yang X Man J He Q Wu Q. Research Trends on the Relationship Between Microbiota and Gastric Cancer: A Bibliometric Analysis From 2000 to 2019. J Cancer (2020) 11:4823–31. 10.7150/jca.44126 PMC7330707 32626529

[24] Li S Wang H Zheng H Li N Sun C Meng X. Bibliometric Analysis of Pediatric Liver Transplantation Research in PubMed From 2014 to 2018. Med Sci Monit (2020) 26:e922517. 10.12659/MSM.922517 32493895 PMC7294844

[25] Mareev VY Orlova YA Pavlikova EP Akopyan ZA Matskeplishvili ST Plisyk AG. Proactive Anti-Inflammatory and Anticoagulant Therapy in the Treatment of Advanced Stages of Novel Coronavirus Infection (COVID-19). Case Series and Study Design: COLchicine *Versus* Ruxolitinib and Secukinumab in Open Prospective Randomized Trial (COLORIT). Kardiologiia (2020) 60:4–21. 10.18087/cardio.2020.9.n1338 33131470

[26] Caviezel C Weiss L Haessig G Alfaré C Haberecker M Varga Z. Case Report of Sequential Bilateral Spontaneous Pneumothorax in a Never-Ventilated, Lung-Healthy COVID-19-Patient. Int J Surg Case Rep (2020) 75:441–5. 10.1016/j.ijscr.2020.09.148 PMC7527682 33076191

[27] Ackermann M Verleden SE Kuehnel M Haverich A Welte T Laenger F. Pulmonary Vascular Endothelialitis, Thrombosis, and Angiogenesis in Covid-19. New Engl J Med (2020) 383:120–8. 10.1056/NEJMoa2015432 PMC7412750 32437596

[28] Scholkmann F Nicholls J. Pulmonary Vascular Pathology in Covid-19. New Engl J Med (2020) 383:887–8. 10.1056/NEJMc2022068 32678533

[29] Yang ZL Chen C Huang L Zhou SC Hu YN Xia LM. Fibrotic Changes Depicted by Thin-Section CT in Patients With COVID-19 at the Early Recovery Stage: Preliminary Experience. Front Med (2020) 7:605088. 10.3389/fmed.2020.605088 PMC7732534 33330571

[30] Ilic I Zdravkovic M Timcic S Stojanovic DU Bojic M Loncar G. Pneumonia in Medical Professionals During COVID-19 Outbreak in Cardiovascular Hospital. Int J Infect Dis (2020) 103:188–93. 10.1016/j.ijid.2020.11.156. PMC7674984 33220441

[31] Varshney AS Wang DE Bhatt AS Blood A Sharkawi MA Siddiqi HK. Characteristics of Clinical Trials Evaluating Cardiovascular Therapies for Coronavirus Disease 2019 Registered on ClinicalTrials.gov: A Cross Sectional Analysis. Am Heart J (2020) 232:105–15. 10.1016/j.ahj.2020.10.065. PMC7586939 33121978

[32] Shi S Qin M Shen B Cai Y Liu T Yang F. Association of Cardiac Injury With Mortality in Hospitalized Patients With COVID-19 in Wuhan, China. JAMA Cardiol (2020) 5:802–10. 10.1001/jamacardio.2020.0950 PMC7097841 32211816

[33] Li X Pan X Li Y An N Xing Y Yang F. Cardiac Injury Associated With Severe Disease or ICU Admission and Death in Hospitalized Patients With COVID-19: A Meta-Analysis and Systematic Review. Crit Care (London England) (2020) 24:468. 10.1186/s13054-020-03183-z PMC7386170 32723362

[34] Nabors C Sridhar A Hooda U Lobo SA Levine A Frishman WH. Characteristics and Outcomes of Patients 80 Years and Older Hospitalized With Coronavirus Disease 2019 (COVID-19). Cardiol Rev (2020) 29(1):39–42. 10.1097/CRD.00000000000. 33136582

[35] Singhavi R Sharma K Desai HD Patel R Jadeja D. A Case of Hemolytic Anemia With Acute Myocarditis and Cardiogenic Shock: A Rare Presentation of COVID-19. Cureus (2020) 12:e10657. 10.7759/cureus.10657 33133827 PMC7587209

[36] Keri VC Hooda A Kodan P Brunda RL Jorwal P Wig N. Intricate Interplay Between Covid-19 and Cardiovascular Diseases. Rev Med Virol (2020) 31(4):e2188. 10.1002/rmv.2188. 33128859

[37] Wen W Zhang H Zhou M Cheng Y Ye L Chen J. Arrhythmia in Patients With Severe Coronavirus Disease (COVID-19): A Meta-Analysis. Eur Rev Med Pharmacol Sci (2020) 24:11395–401. 10.26355/eurrev_202011_23632 33215461

[38] Ziegler CGK Allon SJ Nyquist SK Mbano IM Miao VN Tzouanas CN. SARS-CoV-2 Receptor ACE2 Is an Interferon-Stimulated Gene in Human Airway Epithelial Cells and Is Detected in Specific Cell Subsets Across Tissues. Cell (2020) 181:1016–1035.e19. 10.1016/j.cell.2020.04.035 32413319 PMC7252096

[39] Guo J Wei X Li Q Li L Yang Z Shi Y. Single-Cell RNA Analysis on ACE2 Expression Provides Insights Into SARS-CoV-2 Potential Entry Into the Bloodstream and Heart Injury. J Cell Physiol (2020) 235:9884–94. 10.1002/jcp.29802 PMC7301022 32510598

[40] Reynolds HR Adhikari S Pulgarin C Troxel AB Iturrate E Johnson SB. Renin-Angiotensin-Aldosterone System Inhibitors and Risk of Covid-19. New Engl J Med (2020) 382:2441–8. 10.1056/NEJMoa2008975 PMC7206932 32356628

[41] Fried JA Ramasubbu K Bhatt R Topkara VK Clerkin KJ Horn E. The Variety of Cardiovascular Presentations of COVID-19. Circulation (2020) 141:1930–6. 10.1161/CIRCULATIONAHA.120.047164 PMC7314498 32243205

[42] Puntmann VO Carerj ML Wieters I Fahim M Arendt C Hoffmann J. Outcomes of Cardiovascular Magnetic Resonance Imaging in Patients Recently Recovered From Coronavirus Disease 2019 (COVID-19). JAMA Cardiol (2020) 5:1265–73. 10.1001/jamacardio.2020.3557 PMC7385689 32730619

[43] Koralnik IJ Tyler KL. COVID-19: A Global Threat to the Nervous System. Ann Neurol (2020) 88:1–11. 10.1002/ana.25807 32506549 PMC7300753

[44] Li YC Bai WZ Hashikawa T. The Neuroinvasive Potential of SARS-CoV2 may Play a Role in the Respiratory Failure of COVID-19 Patients. J Med Virol (2020) 92:552–5. 10.1002/jmv.25728 PMC7228394 32104915

[45] Hussain S Vattoth S Haroon KH Muhammad A. A Case of Coronavirus Disease 2019 Presenting With Seizures Secondary to Cerebral Venous Sinus Thrombosis. Case Rep Neurol (2020) 12:260–5. 10.1159/000509505 PMC7490494 33078062

[46] Amouri J Andrews PS Heckers S Ely EW Wilson JE. A Case of Concurrent Delirium and Catatonia in a Woman With Coronavirus Disease 2019. Psychosomatics (2020) 62(1):109–14. 10.1016/j.psym.2020.09.002 PMC7491455 33069380

[47] Braga LFB Sass N. Coronavirus 2019, Thrombocytopenia and HELLP Syndrome: Association or Coincidence? Rev Bras Ginecol Obstet (2020) 42:669–71. 10.1055/s-0040-1718437 33129222

[48] Carrillo-Larco RM Altez-Fernandez C Ravaglia S Vizcarra JA. COVID-19 and Guillain-Barre Syndrome: A Systematic Review of Case Reports. Wellcome Open Res (2020) 5:107. 32995555 10.12688/wellcomeopenres.15987.1 PMC7509591

[49] Rajdev K Victor N Buckholtz ES Hariharan P Saeed MA Hershberger DM. A Case of Guillain-Barré Syndrome Associated With COVID-19. J Invest Med High Impact Case Rep (2020) 8:2324709620961198. 10.1177/2324709620961198 PMC7545753 32981333

[50] Korem S Gandhi H. Guillain-Barré Syndrome Associated With COVID-19 Disease. BMJ Case Reports# (2020) 13:e237215. 10.1136/bcr-2020-237215 10.1136/bcr-2020-237215 PMC7507330 32958554

[51] Vandervorst F Guldolf K Peeters I Vanderhasselt T Michiels K Berends KJ. Encephalitis Associated With the SARS-CoV-2 Virus: A Case Report. Interdiscip Neurosurge: Adv Techniques Case Manage (2020) 22:100821. 10.1016/j.inat.2020.100821 PMC7347479 32835017

[52] Hirano T Murakami M. COVID-19: A New Virus, But a Familiar Receptor and Cytokine Release Syndrome. Immunity (2020) 52:731–3. 10.1016/j.immuni.2020.04.003 PMC7175868 32325025

[53] Esposito G Pesce M Seguella L Sanseverino W Lu J Sarnelli G. Can the Enteric Nervous System be an Alternative Entrance Door in SARS-CoV2 Neuroinvasion? Brain Behav Immun (2020) 87:93–4. 10.1016/j.bbi.2020.04.060 PMC7179488 32335192

[54] Espíndola OM Brandão CO Gomes YCP Siqueira M Soares CN Lima M. Cerebrospinal Fluid Findings in Neurological Diseases Associated With COVID-19 and Insights Into Mechanisms of Disease Development. Int J Infect Dis (2020) 102:155–62. 10.1016/j.ijid.2020.10.044. PMC7591319 33127503

[55] Keller E Brandi G Winklhofer S Imbach LL Kirschenbaum D Frontzek K. Large and Small Cerebral Vessel Involvement in Severe COVID-19: Detailed Clinical Workup of a Case Series. Stroke (2020) 51:Strokeaha120031224. 10.1161/STROKEAHA.120.031224 PMC7678671 33054673

[56] Lechien JR Journe F Hans S Chiesa-Estomba CM Mustin V Beckers E. Severity of Anosmia as an Early Symptom of COVID-19 Infection May Predict Lasting Loss of Smell. Front Med (2020) 7:582802. 10.3389/fmed.2020.582802 PMC7732577 33330539

[57] Eslami P Moradi M Dooghaie Moghadam A Pirsalehi A Abdul Lateef S Hadaegh A. Lethal Outcome of Covid-19 Pneumonia in a New Liver Recipient With Neurological Manifestation. Gastroenterol Hepatol Bed To Bench (2020) 13:405–9. 10.3174/ajnr.A6961 PMC7682977 33244386

[58] Li MD Lang M. Analysis of Stroke Detection During the COVID-19 Pandemic Using Natural Language Processing of Radiology Reports. Am J Neuroradiol (2020) 42:429–34. 10.3174/ajnr.A6961. PMC7959438 33334851

[59] Zhong P Xu J Yang D Shen Y Wang L Feng Y. COVID-19-Associated Gastrointestinal and Liver Injury: Clinical Features and Potential Mechanisms. Sig Trans Targ Ther (2020) 5:256. 10.1038/s41392-020-00373-7 PMC7605138 33139693

[60] Huang C Wang Y Li X Ren L Zhao J Hu Y. Clinical Features of Patients Infected With 2019 Novel Coronavirus in Wuhan, China. Lancet (London England) (2020) 395(10223):497–506. 10.1016/S0140-6736(20)30183-5. PMC7159299 31986264

[61] Chen F Chen W Chen J Xu D Xie W Wang X. Clinical Features and Risk Factors of COVID-19-Associated Liver Injury and Function: A Retrospective Analysis of 830 Cases. Ann Hepatol (2020) 21:100267. 10.1016/j.aohep.2020.09.011. 33053426 PMC7548062

[62] Hammoud S Wehbe Z Abdelhady S Kobeissy F Eid AH El-Yazbi AF. Dysregulation of ACE2 Expression and Function in Co-Morbid Disease Conditions Possibly Contributes to COVID-19 Complication Severity. Mol Pharmacol (2020) 99(1):17–28. 10.1124/molpharm.120.000119. 33082267

[63] Onabajo OO Banday AR Stanifer ML. Interferons and Viruses Induce a Novel Truncated ACE2 Isoform and Not the Full-Length SARS-CoV-2 Receptor. Nat Genet (2020) 52:1283–93. 10.1038/s41588-020-00731-9. PMC9377523 33077916

[64] d’Ettorre G Ceccarelli G Marazzato M Campagna G Pinacchio C Alessandri F. Challenges in the Management of SARS-CoV2 Infection: The Role of Oral Bacteriotherapy as Complementary Therapeutic Strategy to Avoid the Progression of COVID-19. Front Med (2020) 7:389. 10.3389/fmed.2020.00389 PMC7358304 32733907

[65] Scarpellini E Fagoonee S. Gut Microbiota and Liver Interaction Through Immune System Cross-Talk: A Comprehensive Review at the Time of the SARS-CoV-2 Pandemic. J Clinical Med (2020) 9:2488. 10.3390/jcm9082488. PMC7464500 32756323

[66] Stavropoulou E Bezirtzoglou E. Probiotics in Medicine: A Long Debate. Front Immunol (2020) 11:2192. 10.3389/fimmu.2020.02192 33072084 PMC7544950

[67] Han X Ye Q. Kidney Involvement in COVID-19 and Its Treatments. J Med Virol (2020) 93:1387–95. 67. 10.1002/jmv.26653. 33150973

[68] Nadim MK Forni LG. COVID-19-Associated Acute Kidney Injury: Consensus Report of the 25th Acute Disease Quality Initiative (ADQI) Workgroup. Nat Rev Nephrol (2020) p. 1–18. 10.1038/s41581-020-00356-5 33060844 PMC7561246

[69] Ronco C Reis T Husain-Syed F. Management of Acute Kidney Injury in Patients With COVID-19. Lancet Respir Med (2020) 8:738–42. 10.1016/S2213-2600(20)30229-0 PMC7255232 32416769

[70] Guan WJ Ni ZY Hu Y Liang WH Ou CQ He JX. Clinical Characteristics of Coronavirus Disease 2019 in China. N Engl J Med (2020) 382:1708–20. 10.1056/NEJMoa2002032 PMC7092819 32109013

[71] Wang D Hu B Hu C Zhu F Liu X Zhang J. Clinical Characteristics of 138 Hospitalized Patients With 2019 Novel Coronavirus-Infected Pneumonia in Wuhan, China. Jama (2020) 323:1061–9. 10.1001/jama.2020.1585 PMC7042881 32031570

[72] Yang X Yu Y Xu J Shu H Xia J Liu H. Clinical Course and Outcomes of Critically Ill Patients With SARS-CoV-2 Pneumonia in Wuhan, China: A Single-Centered, Retrospective, Observational Study. Lancet Respir Med (2020) 8:475–81. 10.1016/S2213-2600(20)30079-5 PMC7102538 32105632

[73] Kolhe NV Fluck RJ Selby NM Taal MW. Acute Kidney Injury Associated With COVID-19: A Retrospective Cohort Study. PLoS Med (2020) 17:e1003406. 10.1371/journal.pmed.1003406 33125416 PMC7598516

[74] Santoriello D Khairallah P Bomback AS Xu K Kudose S. Postmortem Kidney Pathology Findings in Patients With COVID-19. J Am Soc Nephrol : JASN (2020) 31:2158–67. 10.1681/ASN.2020050744 PMC7461662 32727719

[75] Parr SK Matheny ME Abdel-Kader K Greevy RA Jr. Bian A Fly J. Acute Kidney Injury Is a Risk Factor for Subsequent Proteinuria. Kidney Int (2018) 93:460–9. 10.1016/j.kint.2017.07.007 PMC5796522 28927644

[76] Herrera-Valdés R Almaguer-López M López-Marín L Bacallao-Méndez R Jorge F Guerra-Bustillo G. COVID-19 and the Kidneys: Risk, Damage and Sequelae. MEDICC Rev (2020) 22:87–8. 10.37757/MR2020.V22.N4.10 33295327

[77] Parmar MS. Acute Kidney Injury Associated With COVID-19 - Cumulative Evidence and Rationale Supporting Against Direct Kidney Injury (Infection). Nephrol (Carlton Vic.) (2020) 26(3):239–47. 10.1111/nep.13814. 33150674

[78] Ahmadian E Hosseiniyan Khatibi SM Razi Soofiyani S Abediazar S Shoja MM Ardalan M. Covid-19 and Kidney Injury: Pathophysiology and Molecular Mechanisms. Rev Med Virol (2020) p. e2176. 10.1002/rmv.2176 33022818 PMC7646060

[79] Lundholm MD Poku C. SARS-CoV-2 (COVID-19) and the Endocrine System. J Endocrine Soc (2020) 4:bvaa144. 10.1210/jendso/bvaa144 33145472 PMC7543511

[80] Singh AK Gupta R Ghosh A Misra A. Diabetes in COVID-19: Prevalence, Pathophysiology, Prognosis and Practical Considerations. Diabetes Metab Syndrome (2020) 14:303–10. 10.1016/j.dsx.2020.04.004 PMC7195120 32298981

[81] Abdi A Jalilian M Sarbarzeh PA Vlaisavljevic Z. Diabetes and COVID-19: A Systematic Review on the Current Evidences. Diabetes Res Clin Pract (2020) 166:108347. 10.1016/j.diabres.2020.108347 32711003 PMC7375314

[82] Gupta N Ish P Kumar R Dev N Yadav SR Malhotra N. Evaluation of the Clinical Profile, Laboratory Parameters and Outcome of Two Hundred COVID-19 Patients From a Tertiary Centre in India. Monaldi Arch Chest Dis (2020) 90. 10.4081/monaldi.2020.1507 33169598

[83] Peng X Chen Y Deng L Liu Q Li Q Xiong J. Clinical Features of Critically Ill Patients Infected With SARS-CoV-2 Outside Wuhan With and Without Diabetes. Int J Diabetes Develop Countries (2020) p. 1–9. 10.1007/s13410-020-00888-3 PMC7642858 33169053

[84] Freire Santana M Borba MGS Baía-da-Silva DC Val F Alexandre MAA Brito-Sousa JD. Case Report: Adrenal Pathology Findings in Severe COVID-19: An Autopsy Study. Am J Trop Med Hygien (2020) 103:1604–7. 10.4269/ajtmh.20-0787 PMC7543860 32876012

[85] Iuga AC Marboe CC Yilmaz MM Lefkowitch JH Gauran C Lagana SM. Adrenal Vascular Changes in COVID-19 Autopsies. Arch Pathol Lab Med (2020) 144:1159–60. 10.5858/arpa.2020-0248-LE 32579380

[86] Lui DTW Lee CH Chow WS Lee ACH Tam AR Fong CHY. Thyroid Dysfunction in Relation to Immune Profile, Disease Status and Outcome in 191 Patients With COVID-19. J Clin Endocrinol Metab (2020) 106(2):e926–35. 10.1210/clinem/dgaa813. PMC7665541 33141191

[87] Ruano R Zorzano-Martinez M Campos A Rius F Hernández M. Subacute Thyroiditis Might Be a Complication Triggered by SARS-CoV-2. Endocrinol Diabetes y Nutricion (2020) S2530-0164(20)30206–8. 10.1016/j.endinu.2020.09.002. 10.1016/j.endinu.2020.09.002 PMC7553120 33139217

[88] Zou R Wu C Zhang S Wang G Zhang Q Yu B. Euthyroid Sick Syndrome in Patients With COVID-19. Front Endocrinol (2020) 11:566439. 10.3389/fendo.2020.566439 PMC7575767 33117282

[89] Raghavan D Tan AR Story ES Burgess EF Musselwhite L Kim ES. Management Changes for Patients With Endocrine-Related Cancers in the COVID-19 Pandemic. Endocrine-Related Cancer (2020) 27:R357–r374. 10.1530/ERC-20-0229 32744242

[90] Vishvkarma R Rajender S. Could SARS-CoV-2 Affect Male Fertility? Andrologia (2020) 52:e13712. 10.1111/and.13712 32578263 PMC7361071

[91] Ren X Wang S Chen X Wei X Li G Ren S. Multiple Expression Assessments of ACE2 and TMPRSS2 SARS-CoV-2 Entry Molecules in the Urinary Tract and Their Associations With Clinical Manifestations of COVID-19. Infect Drug Resist (2020) 13:3977–90. 10.2147/IDR.S270543 PMC7650837 33177848

[92] Corona G Baldi E. SARS-CoV-2 Infection, Male Fertility and Sperm Cryopreservation: A Position Statement of the Italian Society of Andrology and Sexual Medicine (SIAMS) (Società Italiana Di Andrologia E Medicina Della Sessualità). J Endocrinol Invest (2020) 43:1153–7. 10.1007/s40618-020-01290-w PMC7252417 32462316

[93] Li H Xiao X Zhang J Zafar MI Wu C Long Y. Impaired Spermatogenesis in COVID-19 Patients. EClinicalMedicine (2020) 28:100604. 10.1016/j.eclinm.2020.100604 33134901 PMC7584442

[94] Pan F Xiao X Guo J Song Y Li H Patel DP. No Evidence of Severe Acute Respiratory Syndrome-Coronavirus 2 in Semen of Males Recovering From Coronavirus Disease 2019. Fertil Steril (2020) 113:1135–9. 10.1016/j.fertnstert.2020.04.024 PMC7164916 32482249

[95] Ali FF Ahmed AF Elroby Ali DM. Underlying Mechanisms Behind the Protective Effect of Angiotensin (1-7) in Experimental Rat Model of Ovarian Ischemia Reperfusion Injury. Life Sci (2019) 235:116840. 10.1016/j.lfs.2019.116840 31494171

[96] Reis FM Bouissou DR Pereira VM Camargos AF dos Reis AM Santos RA. Angiotensin-(1-7), its Receptor Mas, and the Angiotensin-Converting Enzyme Type 2 are Expressed in the Human Ovary. Fertil Steril (2011) 95:176–81. 10.1016/j.fertnstert.2010.06.060 20674894

[97] Jing Y Run-Qian L Hao-Ran W Hao-Ran C Ya-Bin L Yang G. Potential Influence of COVID-19/ACE2 on the Female Reproductive System. Mol Hum Reprod (2020) 26:367–73. 10.1093/molehr/gaaa030 PMC7239105 32365180

[98] Zupin L Pascolo L Zito G Ricci G Crovella S. SARS-CoV-2 and the Next Generations: Which Impact on Reproductive Tissues? J Assist Reprod Genet (2020) 37:2399–403. 10.1007/s10815-020-01917-0 PMC7419027 32783136

[99] Disser NP De Micheli AJ Schonk MM Konnaris MA Piacentini AN Edon DL. Musculoskeletal Consequences of COVID-19. J Bone Joint Surg Am (2020) 102:1197–204. 10.2106/JBJS.20.00847 PMC7508274 32675661

[100] Gonzalez A Orozco-Aguilar J. SARS-CoV-2/Renin-Angiotensin System: Deciphering the Clues for a Couple With Potentially Harmful Effects on Skeletal Muscle. Intern J Mol Sci (2020) 21:7904. 10.3390/ijms21217904. PMC7663203 33114359

[101] Salvio G Gianfelice C Firmani F Lunetti S Balercia G Giacchetti G. Bone Metabolism in SARS-CoV-2 Disease: Possible Osteoimmunology and Gender Implications. Clin Rev Bone Min Metabol (2020), 1–7. 10.1007/s12018-020-09274-3 PMC7459260 32904892

[102] Nakamura K Saito K Hara Y Aoyagi T Kitakawa K Abe Y. Severe Epidemic Myalgia With an Elevated Level of Serum Interleukin-6 Caused by Human Parechovirus Type 3: A Case Report and Brief Review of the Literature. BMC Infect Dis (2018) 18:381. 10.1186/s12879-018-3284-5 30086720 PMC6081802

[103] Roschel H Artioli GG Gualano B. Risk of Increased Physical Inactivity During COVID-19 Outbreak in Older People: A Call for Actions. J Am Geriatr Soc (2020) 68:1126–8. 10.1111/jgs.16550 32392620

[104] Tang C Wang Y Lv H Guan Z Gu J. Caution Against Corticosteroid-Based COVID-19 Treatment. Lancet (London England) (2020) 395:1759–60. 10.1016/S0140-6736(20)30749-2 PMC7247780 32464115

[105] Gloster AT Lamnisos D Lubenko J. Impact of COVID-19 Pandemic on Mental Health: An International Study. PloS One (2020) 15:e0244809. 10.1371/journal.pone.0244809 33382859 PMC7774914

[106] Xiong Q Xu M Li J Liu Y Zhang J Xu Y. Clinical Sequelae of COVID-19 Survivors in Wuhan, China: A Single-Centre Longitudinal Study. Clin Microbiol Infect (2021) 27:89–95. 10.1016/j.cmi.2020.09.023 32979574 PMC7510771

[107] Moreno Fernández-Ayala DJ Navas P López-Lluch G. Age-Related Mitochondrial Dysfunction as a Key Factor in COVID-19 Disease. Exp Gerontol (2020) 142:111147. 10.1016/j.exger.2020.111147 33171276 PMC7648491

[108] Boengler K Kosiol M Mayr M Schulz R Rohrbach S. Mitochondria and Ageing: Role in Heart, Skeletal Muscle and Adipose Tissue. J Cachexia Sarcopenia Muscle (2017) 8:349–69. 10.1002/jcsm.12178 PMC5476857 28432755

[109] Asch DA Islam MN Sheils NE Chen Y Doshi JA Buresh J. Patient and Hospital Factors Associated With Differences in Mortality Rates Among Black and White US Medicare Beneficiaries Hospitalized With COVID-19 Infection. JAMA Net Open (2021) 4:e2112842. 10.1001/jamanetworkopen.2021.12842 PMC11849740 34137829

[110] Price-Haywood EG Burton J Fort D Seoane L. Hospitalization and Mortality Among Black Patients and White Patients With Covid-19. New Engl J Med (2020) 382:2534–43. 10.1056/NEJMsa2011686 PMC7269015 32459916

[111] Saatci D Ranger TA Garriga C Clift AK Zaccardi F Tan PS. Association Between Race and COVID-19 Outcomes Among 2.6 Million Children in England. JAMA Pediatr (2021) p. e211685. 10.1001/jamapediatrics.2021.1685. PMC8218232 34152371

