# Supplementary figures and images for: Complication and Sequelae of COVID-19: What Should We Pay Attention to in the Post-Epidemic Era
Source: Front Immunol. 2021 Sep 3;12:711741. doi: 10.3389/fimmu.2021.711741 (PMC8446426; doi:10.3389/fimmu.2021.711741)

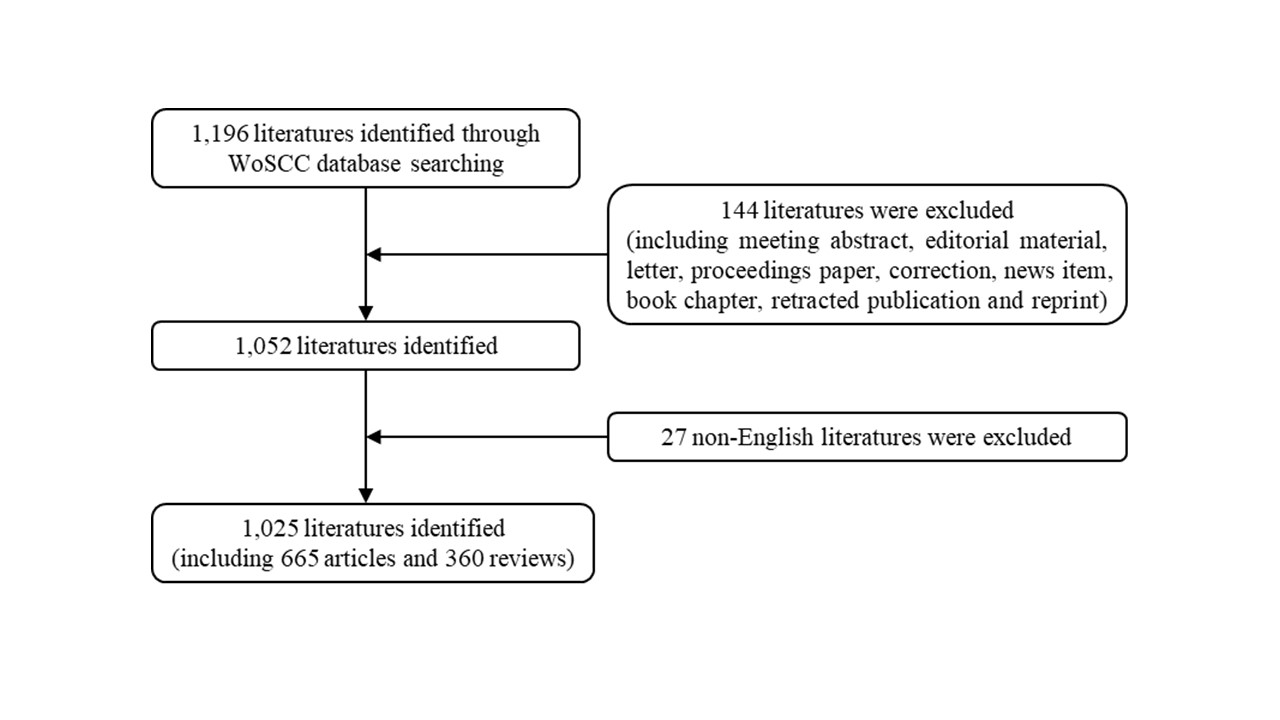

Supplement: Supplementary Figure 1 — Flow chart of literature filtering involved in this study. [file Image_1.jpeg]

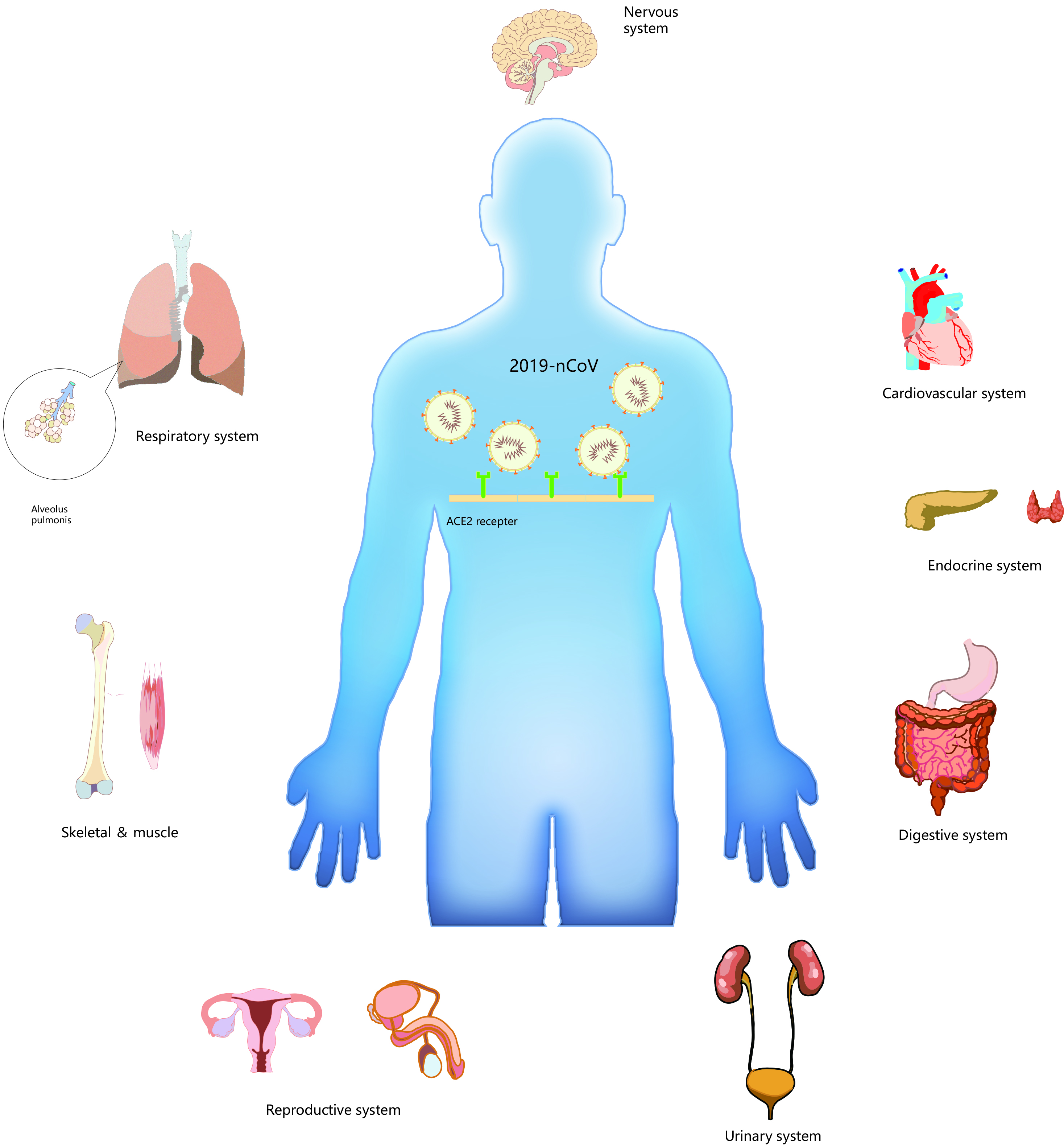

Supplement: Supplementary Figure 2 — Complication and sequelae of COVID-19 patients involved in eight major systems. [file Image_2.jpeg]
